# Bioprospecting endophytic fungi from *Fusarium* genus as sources of bioactive metabolites

**DOI:** 10.1080/21501203.2019.1645053

**Published:** 2019-07-31

**Authors:** Rufin Marie Kouipou Toghueo

**Affiliations:** Antimicrobial and Biocontrol Agents Unit (AmBcAU), Laboratory for Phytobiochemistry and Medicinal Plants Studies, Department of Biochemistry, Faculty of Science, University of Yaoundé I, Yaoundé, Cameroon

**Keywords:** Endophytic fungi, *Fusarium* species, secondary metabolites, biological activities

## Abstract

Endophytic fungi became an attractive source for the discovery of new leads, because of the complexity and the structural diversity of their secondary metabolites. The genus *Fusarium* comprising about 70 species is extremely variable in terms of genetics, biology, ecology, and consequently, secondary metabolism and have been isolated from countless plants genera from diverse habitats. These endophytic microbes may provide protection and survival strategies in their host plants with production of a repertoire of chemically diverse and structurally unprecedented secondary metabolites reported to exhibit an incredible array of biological activities including antimicrobial, anticancer, antiviral, antioxidants, antiparasitics, immunosuppressants, immunomodulatory, antithrombotic, and biocontrol ability against plants pathogens and nematodes. This review comprehensively highlights over the period 1981–2019, the bioactive potential of metabolites produced by endophytes from *Fusarium* genus.

**Abbreviations**: AIDS: Acquired immune deficiency syndrome; BAPT: C-13 phenylpropanoid side chain-CoA acyltransferase; CaBr2: Calcium bromide; DBAT: 10-deacetylbaccatin III-10-O-acetyl transferase; DNA: Deoxyribonucleic acid; EI-MS: Electron ionization mass spectrometer; EN: Enniatin; ERK: Extracellular regulated protein kinase; EtOAc: Ethyl acetate; FDA: Food and Drug Administration; GAE/g: Gallic acid equivalent per gram; GC-MS: Gas chromatography–mass spectrometry; HA: Hyperactivation; HCV: Hepatitis C Virus; HCVPR: Hepatitis C Virus protease; HeLa: Human cervical cancer cell line; HIV: Human immunodeficiency viruses; HPLC: High Performance Liquid Chromatography; IAA: Indole‐3‐acetic acid; IARC: International Agency for Research on Cancer; IC_50_: Half maximal inhibitory concentration; LC_50_: Concentration of the compound that is lethal for 50% of exposed population; LC-MS: Liquid chromatography–mass spectrometry; MCF-7: Human breast cancer cell line; MDR: Multidrug-resistant; MDRSA: Multidrug-resistant *S. aureus*; MFC: Minimum fungicidal concentration; MIC: Minimum inhibitory concentration; MRSA: Multidrug-resistant *S. aureus*; MTCC: Microbial type culture collection; PBMCs: Peripheral blood mononuclear cells; PCR: Polymerase chain reaction; TB: Tuberculosis; TLC: Thin layer chromatography; TNF: Tumor necrosis factor; WHO: World Health Organization

http://www.zoobank.org/urn:lsid:zoobank.org:pub:D0A7B2D8-5952-436D-85C8-C79EAAD1013C

## Introduction

The enormous impact of infectious diseases on our world today cannot be dramatized   and neither underrated (Fauci and Morens 2012). In fact, today’s world is facing the high-impact of some infectious diseases such as Zika, Ebola, West Nile, influenza, food-borne illness, and global pandemics like HIV, TB, and malaria (Tacconelli et al. 2018) without leaving aside others diseases such as cancers (Plummer et al. 2016) and neurodegenerative disorders (Gitler et al. 2017). The threats from neglected tropical diseases, healthcare-associated infections, and invasive fungal infections, not to mention the continued discovery of new and emerging pathogens is constant (Woolhouse and Dye 2001; Woolhouse and Gowtage-Sequeria 2005; Chiller 2017). To worsen the situation, the antimicrobial resistance (AMR) is increasingly recognized as a public health crisis requiring a global action (Looke et al. 2015). In fact, WHO identified more than 20 of the most important resistant pathogens at a global level for which there is an urgent need for new treatments (Tacconelli et al. 2018).

The recent report of the Food and Drug Administration (FDA) shows that from the 38% of drugs discovered from natural products, microbes contributed to about 25%. These findings have really highlight the critical role of microorganisms as a sustainable pipeline for new drug discovery (Newman and Cragg 2016). Indeed, natural product-derived compounds provided impressive and continuous pools for medicinal chemistry applications and encouraged most of the leading pharmaceutical companies in screening microbial natural extracts for the development of high-throughput libraries (Hung and Lin 2017). Since it is estimated to only 1% the proportion of microbial pathogens (Woolhouse and Gowtage-Sequeria 2005), more than 90% of the existing non-pathogenic microbial communities can be fully explored to identify potential active compounds needed to cure diseases affecting mankind. Microbes are extraordinary organisms with the ability to lives in unusual habitats, including extreme temperature, pressure, acidity, or basicity, or within higher organisms which consequently affect their secondary metabolism (Schulze-Makuch et al. 2018). Living inside the tissues of higher plants, endophytic fungi have gained incredible attention over the past decades because of their great diversity and their exceptional ability to produce structurally novel and complex bioactive metabolites (Strobel 2018). Overall, almost all genera of fungi have been isolated as endophytes of plants, including *Fusarium* species.

With their ability to colonize a large variety of plants species (Imazaki and Kadota 2015), *Fusarium* represents a large cosmopolitan genus comprising more than 70 species capable of producing a wide array of active metabolites (Summerell and Leslie 2011). The extraordinary discrepancy within the genus in terms of genetics, which affect not only their biology and interaction with their surrounding organisms, but also their secondary metabolism has made *Fusarium* species one of the most important group of fungi (Stępień et al. 2018). The facility of colonizing diverse hosts has been credited to their outstanding biosynthetic abilility allowing them to occupied their ecological niche (Bills and Gloer 2016). Any species belonging to *Fusarium* genus, can be isolated as an endophyte of plants. This endophytic lifestyle has therefore given them an ability to produce diverse chemical scaffolds (Kaul et al. 2016). Some of their metabolites are produced by many species, while others are limited to a few or only a single species (Thrane 2001). This secondary metabolism is believed to be a chemical defence mechanism initiated by endophytes to assist the host plant under attack by insects and pathogens (Ji et al. 2009). Overall, these metabolites have been found to display an incredible spectrum of biological activities. As a matter of fact, some of the most important therapeutic agents and lead compounds implicated in the development of effective therapies against diseases such as cancer, malaria, neurological, cardiovascular diseases, and autoimmune disorders have been identified in the metabolome of several *Fusarium* species. Moreover, fungi from this genus have demonstrated their great agricultural importance not only by acting as biocontrol agents, but also by producing fungicidal and nematicidal chemicals. Altogether, this genus is a prolific source of bioactive secondary metabolites and can contribute in a spectacular and indispensable way to improve human health. To shed light upon the ability of endophytic *Fusarium* species to produce such useful metabolites applicable in pharmaceutical and agricultural industries, we summarized in this review the data available in past and recent (1981–2019) literature reports on the bioactive potential of metabolites produced by endophytic belonging to this genus.

## 1. Medicines from Fusarium species

The concept of bioprospecting natural products is well established and recognized, thanks to the number of well-known drugs originated from natural sources. For decades, the drive to investigate microorganisms for potential medical application have been nurtured by the discovery of *Penicillium* sp.-producing penicillin (Fleming 1929). This led to the recognition of plant-derived endophytic fungi as capable of producing such life-changing molecules (Strobel and Daisy 2003; Strobel 2018). Undeniably, endophytic *Fusarium* species living inside host plant tissues without causing any symptoms of disease have proven over the years an outstanding potential by producing compounds actually approved for the treatment of several diseases including cancer, malaria, oxidative stress-related diseases, and inflammatory disorders. In fact, medicines such as vinblastine and vincristine (Zhang et al. 2000; Tung et al. 2002; Kumar et al. 2013; Ashuthosh et al. 2013), podophyllotoxin (Kour et al. 2008; Nadeem et al. 2012), camptothecin and its analogues (Kusari et al. 2009; Shweta et al. 2010; Venugopalan et al. 2016; Ran et al. 2017), taxol (Li et al. 2009; Gurudatt et al. 2010; Elavarasi et al. 2012; Xiong et al. 2013), rohitukine (Kumara et al. 2014), ginkgolide B (Cui et al. 2012), quinine and cinchonidine (Hidayat et al. 2015) have been reported as products of *in vitro* fermentation of several *Fusarium* species. These findings have been over the years a catalyst to continue the exploration of this genus with a goal to sustain the production of such pharmacologically important drugs via microbial fermentation but have also encouraged the investigation of these species as sources of a potential drug candidates against diverse diseases. The Table 1 is summarizing the important drugs identified in the metabolome of *Fusarium* spp., the fungal-producing strain and the yield of production.

**Table 1.** Well-known Drugs produced by *Fusarium* species through *in vitro* fermentation.

Medicines	Producing- fungal	Host-plant	Production yield	References
Quinine and Cinchonidine	*F. incarnatum*, *F. oxysporum*, *F. solani*	*C. calisaya*	0.8–0.9mg/L	Hidayat et al. (2015)
Camptothecin	*F. solani*	*C. acuminata*	150 μg/L	Ran et al. (2017)
		-	28.9 μg/L	Venugopalan et al. (2016)
		*A. dimidiata*	37–53 µg/100 g	Shweta et al. (2010)
		*C. acuminata*	*	Kusari et al. (2009)
	*F. sacchari*	*N. nimmoniana*	*	Gurudatt et al. (2010)
10-hydroxycamptothecin	*F. solani*	*A. dimidiata*	8.2 µg/100 g	Shweta et al. (2010)
		*C. acuminata*	*	Kusari et al. (2009)
9-methoxycamptothecin	*F. solani*	*A. dimidiata*	44.9 µg/100 g	Shweta et al. (2010)
		*C. acuminata*	*	Kusari et al. (2009)
Ginkgolide B	*F. oxysporum*	*G. biloba*	*	Cui et al. (2012)
Podophyllotoxin	*F. oxysporum*	*J. recurva*	28μg/g	Kour et al. (2008)
	*F. solani*	*P. hexandrum*	29.0μg/g	Nadeem et al. (2012)
Rohitukine	*F. oxysporum*	*D. binectariferum*	2.952 μg/g	Kumara et al. (2014)
	*F. oxysporum*	*D. binectariferum*	3.2949μg/g	
	*F. solani*	*D. binectariferum*	3.5955μg/g	
Taxol	*F. oxysporum*	*R. annamalayana*	172.3 μg/L	Elavarasi et al. (2012)
	*F. anthrosporioides*	*T. cuspidata*	131 μg/L	Li et al. (2008)
	*F. lateritium*	*T. buccata*	0.13 μg/L	Strobel et al. (1996)
	*F. mairei*	*T. mairei*	225.2 μg/L	Xu et al. (2006)
	*F. mairei*	*T. chinensis var mairei*	2.72 μg/L	Cheng et al. (2007)
	*F. mairei*	*T. chinensis var mairei*	286.4 μg/L	Dai and Tao (2008)
	*F. mairei*	*T. x media*	20 μg/L	Dai and Tao (2008)
	*F. oxysporum*	*R. annamalayamna*	*	Elavarasi et al. (2012)
	*F. redolens*	*T. buccata*	66.0 μg/L	Garyali et al. (2013)
	*F. solani*	*T. chinensis*	163.35 μg/L	Deng et al. (2009)
	*F. solani*	*T. celebica*	1.6 μg/L	Chakravarthi et al. (2008)
	*F. mairei*	*T. chinensis var. mairei*	25.63 mg/L	Li et al. (2009)
	*F. proliferatum*	*T. media*	240 ng/L	Xiong et al. (2013)
Vinblastine	*F. oxysporum*	*C. roseus*	76 µg/L	Kumar et al. (2013)
Vincristine			67 µg/L	
Vinblastine and vincristine	*F. solani*	*C. roseus*	*	Ashuthosh et al. (2013)

## 2. Exploring fusarium species as sources of antimicrobial agents

Today, the healthcare system is currently being challenged by the emergence and re-emergence of multidrug-resistant (MDR) pathogens. Infections caused by multiresistant pathogens are difficult to cure and the patient must undergo multiple treatments regimen with broad-spectrum antibiotics, known to be less efficient, more toxic and more expensive (Humberto et al. 2010). To address this issue, new antimicrobial compounds are urgently needed in this modern era to fill the drug development pipeline. Several studies have indicated the possible prospect of endophytes from *Fusarium* genus as a promising resource of antimicrobial compounds. In the quest of the potential starting points for the development of new antibiotics, various research groups have investigated the activity of extracts prepared from endophytic fungi belonging to *Fusarium* genus against a broad range of bacteria and fungi pathogens. These studies mostly aimed at identifying potent extracts that could be progressed for chemical analysis in order to isolate and characterise potential active principles. In fact, crude extracts from several fungi including *F. solani* endophyte of *Taxus baccata* (Tayung et al. 2011), *F. equiseti* and *Fusarium* sp. Dzf18 isolated from *Garcinia parvifolia* (Sim et al. 2010), endophytic *F. oxysporum* (Musavi and Balakrishnan 2013), *F. oxysporum* isolated from *C.odorata* (Toghueo et al. 2016a), *F. oxysporum* isolated from *R. apiculate* (Moron et al. 2018) and *F. lateritium* endophyte of *Rhizophora mucronata* (Hamzah et al. 2018) were reported to exhibit a broad antimicrobial spectrum against various pathogenic microorganisms indicating the conceivability of potential bioactive metabolites in their crude mixture. These findings fits well with the general claim that fungi can produce a complex mixture of myriad compounds capable of broad antimicrobial activity. This give rise to further chemical investigation using different chromatographic and spectroscopic tools for both purification and identification of the active ingredients.

This approach so-called bioguided fractionation, has been used to investigate several active extracts from *Fusarium* species and dozens of active compounds were identified (Figure 1). In fact, butanedioic acid (1), 5alpha, 8alpha-epidioxyergosta-6, 22-dien-3beta-ol (2), and, 22-dien-3beta, 5alpha, 6beta, 7alpha-tetraol (3), displaying antimicrobial activity were isolated from active petroleum extract of *Fusarium* sp. Ppf4 endophyte of *P. polyphylla* (Huang et al. 2009). Compounds 3,6,9-trihydroxy-7-methoxy-4,4-dimethyl-3,4-dihydro-1H-benzo[g]isochromene-5,10-dione (4), 3-O-methylfusarubin (5), javanicin (6) and fusarubin (7) exhibiting activity against various bacterial strains (MIC <1 to 256 μg/mL) and *Mycobacterium tuberculosis* strain H37Rv (MIC 8–256 μg/mL) were also isolated from anti-bacterial extract from endophyte *F. solani* (Shah et al. 2017). The extensive chemical investigation of extract from *F. solani* JK10, an endophyte of *Chlorophora regia*, resulted in the isolation of seven new compounds among which compounds 7, 8, 9, 10 and 11 demonstrated antibacterial activity against gram-negative and positive bacteria with MIC 5–10μg/mL (Kyekyeku et al. 2017). Antibacterial compounds, fusarubin (7), bostrycoidin (12), and anhydrofusarubin (13) were also isolated from extract of *F. solani*, endophyte from the root of *C. alata* (Khan et al. 2018). Equisetin (14), the antibacterial compound with MIC 8–16 μg/mL was purified from extract of *Fusarium* sp. isolated from *O. dillenii* by Ratnaweera et al. (2015). Similarly, Jin et al. (2017) reported the activity of ginsenoside (15) (MIC 1.6–3.2 mg/mL) isolated from extract of endophyte *Fusarium* sp. PN8 from *P. notoginseng*.

**Figure 1.**
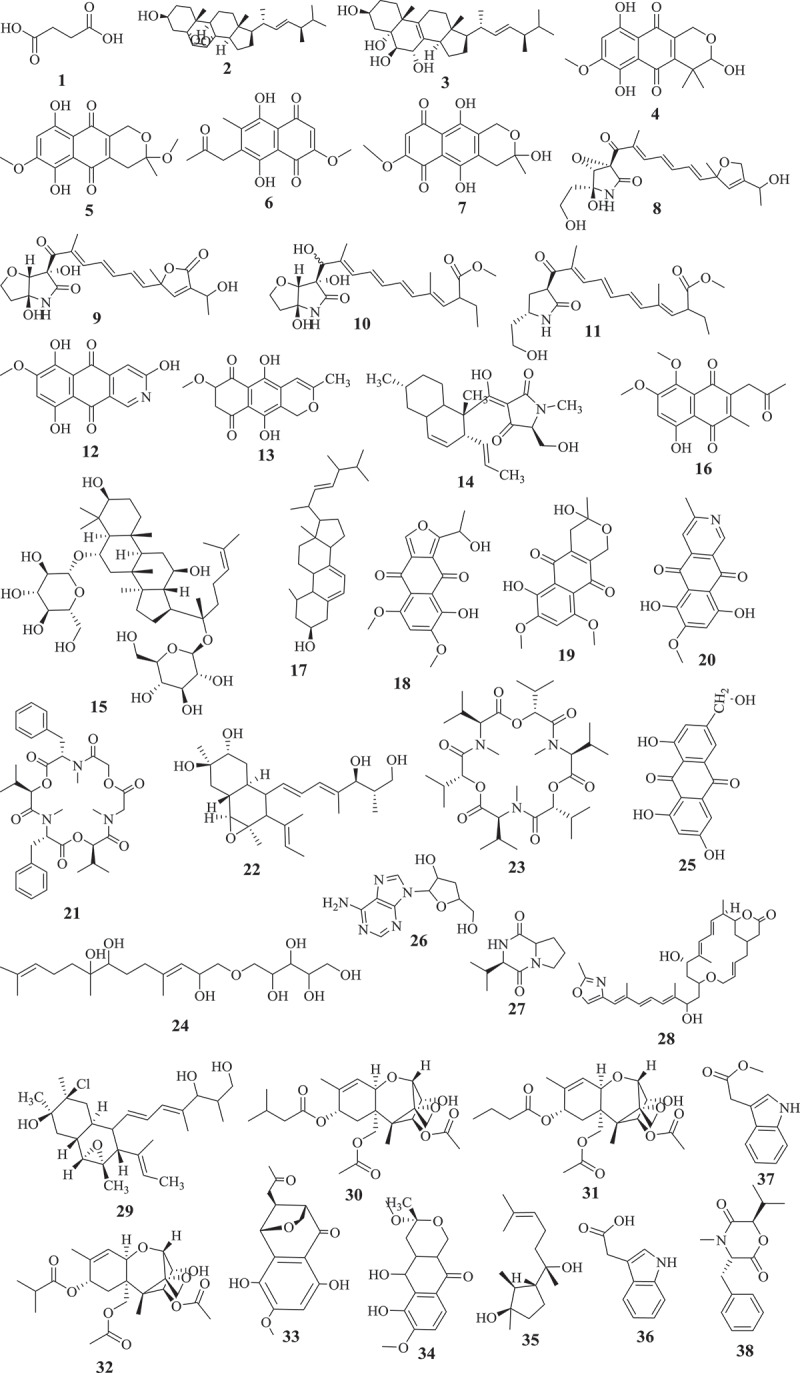
Selected antimicrobial compounds reported from *Fusarium* endophytes.

**Figure 1.**
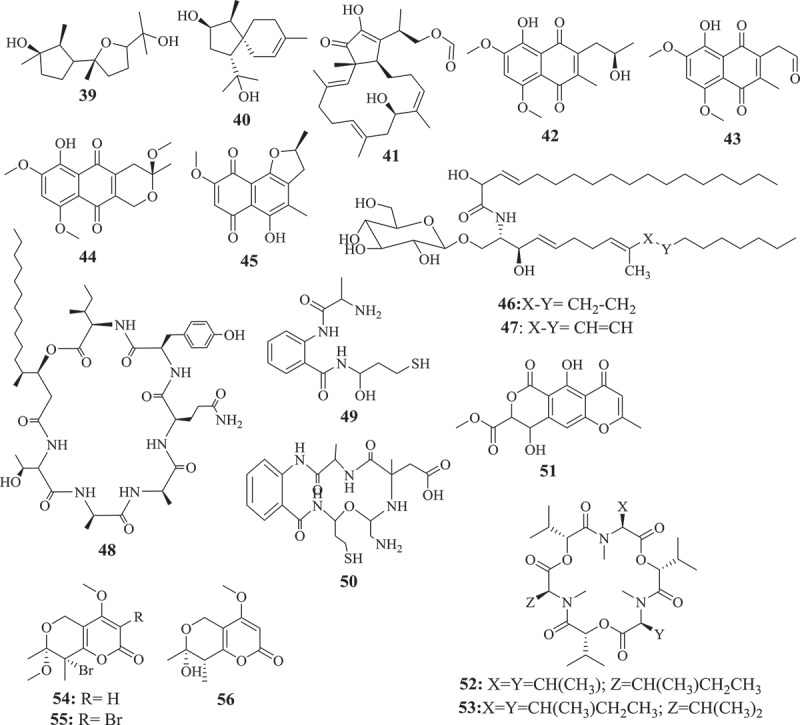
(Continued).

Seven active metabolites, including 8-hydroxy-5,6-dimethoxy-2-methyl-3-(2-oxo-propyl)-1,4-naphthoquinone (16), ergosta-5,7,22-trien-3β-ol (17), nectriafurone-8-methyl ether (18), 9-O-methyl fusarubin (19), bostrycoidin (12), and bostrycoidin-9-methyl ether (20) were isolated from potent extract of *F. proliferatum*, endophyte of *Syzygium cordatum* (Dame et al. 2016). Beauvericin (21) isolated from extracts of endophytes, *F. oxysporum* (*C. kanehirae*) and *F. redolens* Dzf2 (*D. zingiberensis*) were reported to exhibited broad antibacterial activity (Xu et al. 2010; Wang et al. 2011). The bioassay-guided fractionation of *F. tricinctum* extract, an endophyte of *Salicornia bigelovii* led to fusarielin B (22) and enniatin B (23) along with a new sesquiterpenoid, fusartricin (24). All the three compounds were potents against a wide range of pathogens (Zhang et al. 2015a). Among the 20 compounds isolated from ethyl acetate extract of *F. equiseti*, an endophytic fungus isolated from *Padina pavonica*, compounds w-hydroxyemodin (25) and cordycepin (26) were potent against *B. subtilis* and *S. aureus*. Cyclo (D-cis-Hyp-L-Leu) (27) was the most potent against *B. megaterium* while, 17-demethyl-2,11-dideoxy-rhizoxin (28) and w-hydroxyemodin (25) were active against *C. albicans* (Hawas et al. 2016).

The investigation of antifungal extract from *Fusarium* sp. (strain 05JANF165) led to the isolation of a new antifungal compound, fusarielin E (29) (Gai et al. 2007). Similarly, a new pentaketide antifungal agent, CR377, showing potency against *C. albicans* was purified from the extract of *Fusarium* sp. isolated from *Selaginella pallescens* by Brady and Clardy (2000). Another study by Campos et al. (2011) reported the purification of T2‐toxin (30) and a mixture of 8‐n‐butyrylneosolaniol (31) and 8‐isobutyrylsolaniol (32) exhibiting antifungal activity (MIC 75–640μM) against eleven clinical strains of *Paracoccidioides brasiliensis*. Kornsakulkarn et al. (2011) reported that while Javanicin (6) exhibited antifungal activity (IC_50_ 6.16 μg/mL), compounds 5, 6, (1S,4S,10S)-6,9-dihydroxy-8-methoxy-10-(2-oxopropyl)-3,4-dihydro-1,4-methanobenzo[c]oxepin-5(1H)-one (33) and (3R)-5,6-dihydroxy-3,7-dimethoxy-3-methyl-1,3,4,4a,5,10a-hexahydro-10H-benzo[g]isochromen-10-one (34) were active against *Mycobacterium tuberculosis*. Although not highly potent, compounds 33 and 34 are small molecules with structure relatively easy to synthesize through medicinal chemistry efforts. These compounds may offer an opportunity to generate starting points to create a library of semisynthetic compounds needed to help accelerate the discovery of the new antimycobacterial drugs. This can be very useful for drug discovery against tuberculosis since most of the commercial libraries available have already been screen against *M. tuberculosis*. These natural products can provide new scaffolds to boost drug discovery against this infectious disease and many others neglected tropical diseases. Overall, these studies revealed that the exploration of active and extremely complex crude extracts can lead to the purification of unknown compounds exhibiting broad to a narrow spectrum of activity. Dozens of unknown compounds with interesting activity profile were identified through a bioguided exploration of crude metabolites from *Fusarium* spp. This effect-related analytical approach has and continues to be reported as an ideal strategy for exploring natural products for drug discovery. This approach considered as a powerful tool to explore complex mixture to identify a target compounds (Weller 2012) has directed the discovery of countless active compounds from diverse natural sources (Peters et al. 2010; Kildgaard et al. 2017; Bayrami et al. 2018).

Although the bio-guided fractionation is a successful approach to identify potent compounds from crude mixture, several compounds exhibiting interesting activity have also been reported from a chemical investigation of an extract with unknown activity (Figure 1). Therefore, antibacterial (MIC 12.5–100μg/mL) compounds, (1R,2S,3R)-3-((S)-2-hydroxy-6-methylhept-5-en-2-yl)-1,2-dimethylcyclopentan-1-ol (35), indol-3-acetic acid (36), methyl indolyl-3-acetate (37), bassiatin (38), beauvericin (21), epicyclonerodiol oxide (39), 3β-hydroxy-β-acorenol (40), fusaproliferin (41), 5-O-methylsolaniol (42), 5-O-methyljavanicin (43), methyl ether fusarubin (44), and anhydrojavanicin (45) were isolated from extract of *F. proliferatum* AF-04, endophyte of the green Chinese onion (Jiang et al. 2019). Similarly, fusaruside (46), and a known metabolite (2S,2ʹR,3R,3ʹE,4E,8E)-1-O-beta-D-glucopyranosyl-2-N-(2ʹ-hydroxy-3ʹ-octadecenoyl)-3-hydroxy-9-methyl-4,8-sphingadienine (47) both showing strong antibacterial (MIC 1.9–7.8μg/mL) activity were isolated from a chloroform-methanol extract of *Fusarium* sp. IFB-121, an endophytic fungus in *Quercus variabilis* (Shu et al. 2004). Ibrahim et al. (2018a) also reported the strong (MIC 0.11–0.24 μM) antifungal activity of fusaripeptide A (48), a new cyclodepsipeptide isolated from *Fusarium* sp., an endophyte from the roots of *Mentha longifolia* L.

A new benzamide derivative, namely fusarithioamide A (49) with strong antibacterial (MIC ranging from 3.1 to 6.9 μg/mL) was purified from the EtOAc extract of *F. chlamydosporium* isolated from the leaves of *Anvillea garcinii* (Ibrahim et al. 2016a). This finding encourages the same authors to continue the investigation of this extract which led to the identification of fusarithioamide B (50), a new aminobenzamide derivative, exhibiting antimicrobial activity towards *C. albicans, E. coli, B. cereus*, and *S. aureus* with MIC of 1.9 μg/mL (Ibrahim et al. 2018b). The liquid chromatography-mass spectrometry analysis of methanol extract from endophyte *F. tricinctum* isolated from the fruits of *Hordeum sativum* revealed the presence of six antibiotic compounds, enniatins A, A1, B, B1, B2, and Q (Zaher et al. 2014). Even though some active compounds were reported from this approach, this blind investigation of crude extract often led to the isolation of inactive compounds. For instance, Xiao et al. (2018) reported the purification of a novel pyrone derivative bearing two fused five-member rings, together with two new naphthalenone derivatives from an extract of *Fusarium* sp. HP-2, endophytic fungus isolated from “Qi-Nan” agarwood but none of the isolated compounds were activity against a panel of pathogens tested. Likewise, inactive fusarimine, a novel polyketide-derived isoquinoline alkaloid, was isolated from cultures of *Fusarium* sp. LN-12, an endophytic fungus isolated from the leaves of *Melia azedarach* (Yang et al. 2012). *F. sporotrichioides* strain M-1–1 isolated from bean bull was reported to 4 beta, 8 alpha-diacetoxy-12,13-epoxytrichothec-9-ene-3 alpha, 15-diol, and 4 beta-acetoxy-12,13-epoxy-trichothec-9-ene-3 alpha,8 alpha,15-triol (Ishii and Ueno 1981). Similarly, 5-hydroxy-7-methoxy-40-O-(3-methylbut-2-enyl) isoflavone, along with known compounds, eriodictyol, vittarin-B, 3,6,7-trihydroxy-1-methoxyxanthone, 1,3,6-Trihydroxy-8-methylxanthine and cyclo (Phe–Tyr) were isolated from *Fusarium* sp. ZZF60, a mangrove endophytic fungus (Huang et al. 2012). Although, these compounds couldn’t exhibit antimicrobial activity, these data are not enough to classify them as inactive. Further investigation of these compounds against others pathogens (parasites, virus) could reveals outstanding potency.

Apart from the two approaches mentioned above, other strategies such as the coculture or the addition of chemicals to culture medium have been successfully applied to induce the production of active antimicrobial compounds by *Fusarium* spp (Figure 1). In fact, the co-cultivation of endophytic fungal *F. tricinctum* with the *B. subtilis* 168 trpC2 increased by about 78-fold the accumulation of lateropyrone, enniatins, and fusaristatin A. Additional compounds such as (−)-citreoisocoumarin, macrocarpon C, 2-(carboxymethylamino) benzoic acid, and (−)-citreoisocoumarinol were identified. Among these compounds, lateropyrone (51), enniatins B1 (52) and A1 (53), exhibited broad antibacterial activity (Ola et al. 2013). Co-culturing *F. tricinctum* with *S. lividans* also led to the production of new compounds including fusatricinones A–D, and dihydrolateropyrone. In addition, antibiotic compounds such as lateropyrone, enniatins B, B1 and A1, and fusaristatin A, were also upregulated (Moussa et al. 2019).

In addition to co-cultivation, supplementing the culture medium with chemicals has resulted in a production of active compounds (Figure 1). Adding CaBr_2_ to the culture medium of a marine-endophyte *F. tricinctum* resulted in the production of two new compounds, bromomethylchlamydosporols A (54) and B (55), along with chlamydosporol (56) and fusarielin A, all exhibiting activity against sensitive and resistant strains of *S. aureus* (Nenkep et al. 2010). These strategies of inducing new bioactive metabolites are of interest since many secondary metabolism gene clusters are silent under standard laboratory conditions (Bergmann et al. 2007). Therefore, methods such as genetic engineering (Bergmann et al. 2007), mutagenesis, the OSMAC approach (Bode et al. 2002), treatment with epigenetic modifiers (Cichewicz 2010; Brakhage 2013), co-cultivation (Marmann et al. 2014; Bertrand et al. 2014a; Netzker et al. 2015) or addition of small chemicals (Toghueo et al. 2016b, 2018) have been successfully applied during the past years to activate these silent gene clusters in filamentous fungi and induce the formation of many new active metabolites. These strategies have opened new avenues and will certainly continue to play a significant role in the elucidation of cryptic natural products from microbial sources.

## 3. Antiparasitic agents from fusarium species

Among diseases caused by parasites, malaria is the most life-threatening with an estimated number of morbidity cases of 219 million and 435 000 of deaths occurred in 2017, worldwide (WHO 2018). Even though this infection is curable, the global malaria elimination is being challenged by the continued emergence of parasite resistance to antimalarial medicines (WHO 2018). To fill the antimalarial drug development pipeline, new antiplasmodial agents are urgently needed. In the past, the exploration of natural products have provided antimalarial drugs. Therefore, the screening natural sources could lead to the discovery of such lead compounds. In this respect, fusaripeptide A (48) was reported by Ibrahim et al. (2018a) to display significant antiplasmodial activity toward *P. falciparum* (D6 clone) with an IC_50_ value of 0.34 μM (Figure 1). This compound active in the nanomolar range can constitute a great starting point for new drug discovery against malaria.

Although, not many compounds isolated from *Fusarium* spp. have been tested for their antiplasmodial activity, extracts from different *Fusarium* species exhibited very good potency against resistant *P. falciparum* strains. This finding suggests that exploring these species may offer a chance for the discovery of compounds with the potential to cure an infection caused by resistant strains. Indeed, crude metabolites produced by *F. oxysporum* isolated from *Symphonia globulifera* was found to exhibit very good activity (IC_50_ of 1.7µg/mL) against the chloroquine-resistant *P. falciparum* INDO (Ateba et al. 2018). Also, the potency (IC_50_ 1.62–4.38 μg/mL) of extracts from *Fusarium* sp. N240 isolated from *C. odorata* against both resistant and sensitive strains of *P. falciparum* was reported (Toghueo et al. 2018). The similar observation was made while investigating ethyl acetate extract from *Fusarium* sp. AMst1 endophyte of *Annona muricata* against *P. falciparum Pf*3D7, *Pf*Dd2, and *Pf*Indo (IC_50_ 1.16–1.43 μg/mL). This extract was also found to inhibit the transition of the ring to the trophozoite stage (Toghueo et al. 2019). Another study by Kaushik et al. (2014) shows that extract and fractions from endophytic fungus *Fusarium* sp. from a marine alga were very potent against *P. falciparum* 3D7. These studies suggest that deeper bioguided investigations are needed to fully explore the metabolome of these fungi for new antiplasmodial drug discovery.

**Figure 2.**
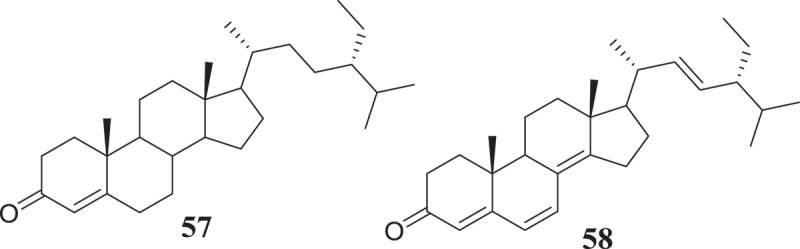
Antitrypanosomal and antileishmanial compounds from *Fusarium* spp.

The potential of endophytic *Fusarium* species to produce compounds active against *Trypanosoma* and *Leishmania* parasites, causative agents of chagas diseases and leishmaniasis, respectively, have been investigated as well (Figure 2). The results reported till now suggest an interesting activity profile and encourage continuing the investigation. As a matter fact, beauvericin (21) isolated from *Fusarium* sp. WC9 was reported to inhibit *T. cruzi* with IC_50_ 2.43 μM (Campos et al. 2015). Against *L. donovani*, the moderate activity of methanol extract of endophyte *F. tricinctum* isolated from the fruits of *Hordeum sativum* was reported by Zaher et al. (2014). The two new compounds, Integracides F (57) and G (58) isolated from *Fusarium* sp., an endophytic fungus of *Mentha longifolia* were found to exhibit anti-leishmanial activity with IC_50_ values of 3.74 and 2.53 μg/mL, respectively (Ibrahim et al. 2016b). Compounds anhydrofusarubin (13) and beauvericin (21) isolated from *F. oxysporum* SS46 endophyte of *Smallanthus sonchifolius* also exhibited promising activity against *Leishmania braziliensis* (Nascimento et al. 2012). Although isolated compounds may be considered as moderate inhibitors, their small molecular-like structure offers the possibility for medicinal chemistry that could improve their drug-likeness and may lead to the discovery of potential drug candidates.

## 4. Endophytic fungi from fusarium species and their antiviral agents

**Figure 3.**
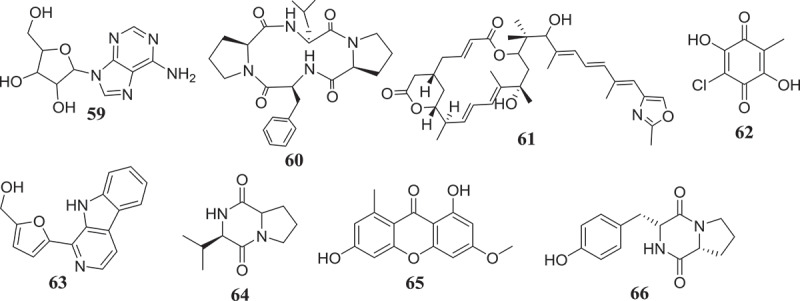
Antiviral compounds from produced by *Fusarium* species.

Over the course of human civilization, viral infections have caused millions of human deaths worldwide, driving the development of antiviral drugs in a pressing need (De Clercq 2004). Endophytic fungi represent a vast reservoir of bioactive molecules, which could potentially be used as antivirals in the future. In this regard, *Fusarium* species have been reported to produce metabolites capable of antiviral activity (Figure 3). The bioguided fractionation of extracts from the endophytic fungus *F. equiseti* led the isolation of several compounds among which cordycepin (36) and ara-A (59) showed less potency, cyclic tetrapeptidecyclo-[Phenylalanyl-pro-leu-pro] (60), 17-demethyl-2,11-dideoxy-rhizoxin (61), 5-chloro-3,6-dihydroxy-2-methyl-1,4-benzoquinone (62) and perlolyrine (63) were moderately potent. Cyclo (L-Pro-L-Val) (64) and griseoxanthone C (65) showed good potency against HCV NS3/4A protease while, ω-hydroxyemodin (25) and cyclo (L-Tyr-L-Pro) (66) were the most potent HCVPR inhibitors (Hawas et al. 2016). Compounds with similar core structure including stachybogrisephenone B, grisephenone A, and 3,6,8-Trihydroxy-1-methylxanthine, isolated from the cultures of sponge-derived fungus *Stachybotry* sp. HH1 ZDDS1F1–2, were also reported to inhibit *in vitro* the replication of EV-71 with IC_50_ values of 30.1, 50.0 and 40.3 μM, respectively (Qin et al. 2014). Similarly, AGI-B4 isolated from marine-derived fungus *Neosartorya fischeri* strain 1008F1 showed strong inhibitory effect on the replication of tobacco mosaic virus (TMV), with IC_50_ values of 260 μM (Tan et al. 2012). The broad antiviral spectrum of these compounds, with similar structure, suggests that further medicinal chemistry efforts may lead to the synthesis of several derivatives among which potential candidates for further studies towards drug discovery against HCV, EV-71, TMV and other related viruses could be identified.

## 5. Endophytic fungi from fusarium species as sources of novel anticancer compounds

**Figure 4.**
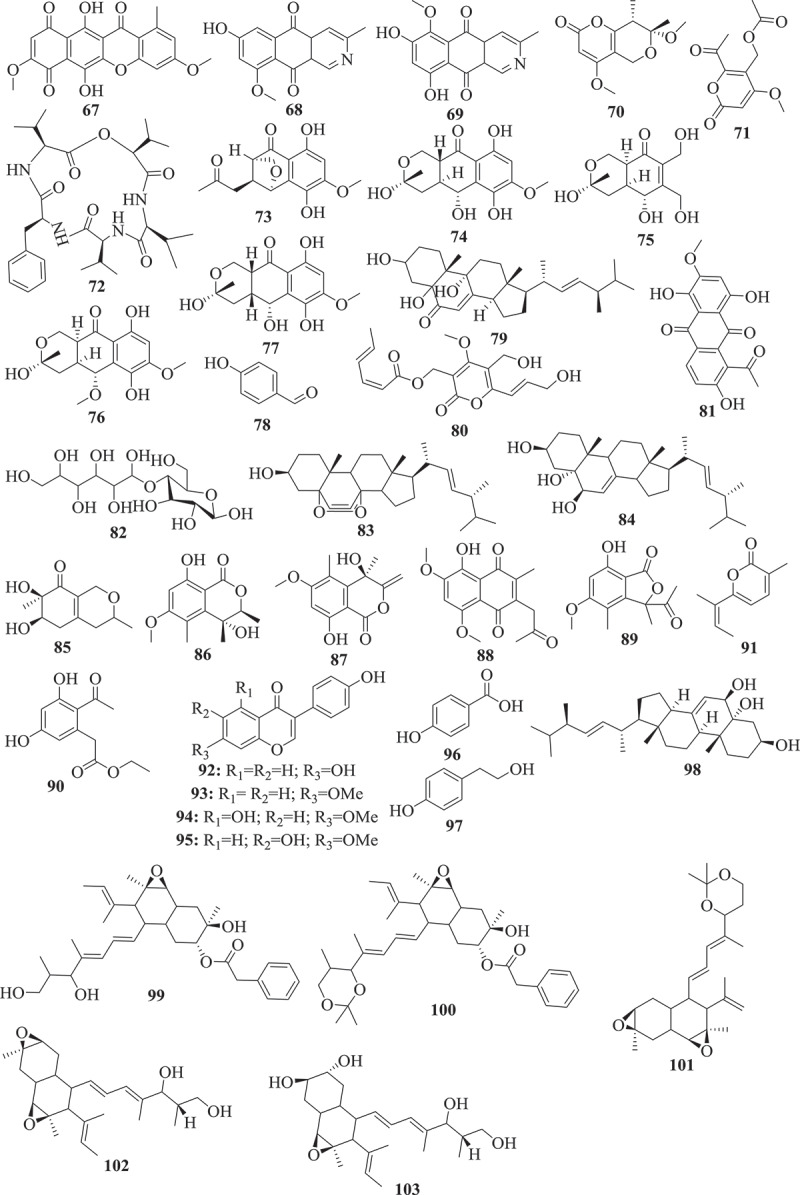
Anticancer compounds isolated from *Fusarium* species.

Since the declaration of the “war on cancer” in 1971 in the United States, only a few battles have been won and the war is still ongoing (Michelakis et al. 2008) and seems endless. With the global cancer burden estimated to have risen to 18.1 million new cases and 9.6 million deaths in 2018, cancer is increasingly risen (IARC Global Cancer Observatory 2018). Despite the seriously challenged breakthrough in anticancer chemotherapy and the endless search for newer anticancer agents, the discovery of endophytic fungi-producing anticancer compounds has given hope to the scientific community (Stierle et al. 1993; Kharwar et al. 2011). Endophytic fungi producing interesting and unmatchable secondary metabolites are very useful in providing chemical scaffolds for anticancer drugs discovery (Tan and Zou 2001). In a continuation of the search for secondary metabolites with anticancer activity, several endophytic fungi strains from *Fusarium* genus have been investigated with promising results (Figure 4).

Using the bioassay-guided investigation, several cytotoxic compounds were identified from endophytic fungi from *Fusarium* species. As a matter of fact, beauvericin (21) and bikaverin (67) among the compounds isolated from EtOAc extract of *F. oxysporum*, endophyte of *Cylindropuntia echinocarpus* were cytotoxic against a panel of four sentinel cancer cell lines, NCI-H460 (non-small-cell lung), MIA Pa Ca-2 (pancreatic), MCF-7 (breast), and SF-268 (CNS glioma). Similarly, Zhan et al. (2007) also showed that among all the compounds isolated from *F. oxysporum* endophyte of *Ephedra fasciculate*, only beauvericin (21) inhibited the metastatic prostate cancer (PC-3M) and breast cancer (MDA-MB-231) cells and also showed antiangiogenic activity in HUVEC-2 cells at sub-lethal concentrations. According to Chowdhury et al. (2017), only 7-desmethylscorpinone (68) and 7-desmethyl-6-methylbostrycoidin (69) among compounds produced by *F. solani* isolated from *Aponogeton undulates* were strongly potent against four human tumour cell lines, MDA MB 231, MIA PaCa2, HeLa, and NCI H1975 (IC_50_ 0.34–30.72µM). Fusarilactone A (70) and fusarilactone B (71) isolated from an extract of endophytic fungus *Fusarium* sp. displayed moderate activity against SMMC-7721, A-549, and MCF-7 cell lines (Chen et al. 2014). Sansalvamide (72), isolated from *Fusarium* sp. collected from *Halodule wrightii* was also found to have significant anticancer activity against the National Cancer Institute’s 60-cell-line panel (Belofsky et al. 1999) and was later identified as an inhibitor of the topoisomerase I by Hwang et al. (1999). Several cytotoxic compounds including dihydronaphthalenone (73), 5-hydroxydihydrofusarubins A (74), 5-hydroxydihydrofusarubins B (75), 5-methoxydihydrofusarubin B (76), and 5-hydroxydihydrofusarubin C (77) were also reported by Kornsakulkarn et al. (2011). Nascimento et al. (2012) after the fractionation of cytotoxic ethyl acetate extract from *F. oxysporum* SS46 identified anhydrofusarubin (13) and beauvericin exhibiting activity against different cancer cell lines. Similarly, compounds 4-hydroxybenzaldehyde (78), bostrycoidin (12), anhydrofusarubin (13) and 3,5,9-trihydroxyergosta-7,22-diene-6-one (79) with significant cytotoxicity against vero cells were isolated from ethyl acetate extract of endophytic fungus *F. solani* by Khan et al. (2018).

Unlike bioguided fractionation, the random investigation of extracts can also lead to the isolation of compounds with potency. In fact, based only on the assumption that fungi belonging to the genus *Fusarium* can produce a cytotoxic metabolites, several compounds were isolated and then tested for their anticancer activity. Allantopyrone A (80) isolated from *Fusarium* sp. IM-37, an endophytic fungus from *R. mucronata* was tested against HL60 cells line and showed activity with IC_50_ of 0.32 mM (Shiono et al. 2014). Integracides F (57) and G (58) was also found to display cytotoxicity towards BT-549 and SKOV-3 (IC_50_ 0.12–1.97µg/mL) (Ibrahim et al. 2016b). Likewise, 5-acetyl-2-methoxy-1,4,6-trihydroxy-anthraquinone (81), isolated from the culture of the endophytic fungus *Fusarium* sp. (No. b77) was cytotoxic against Hep G2 and Hep2 cells (Shao et al. 2010). Several compounds were isolated from extract of *F. equiseti*, an endophyte of *Salicornia bigelovii* and tested for their anticancer activity. Diglucotol (82) and ergosterol peroxide (83) showed weak potency (IC_50_ 52.4–99.39 μM) and cerevisterol (84) was moderately active (IC_50_ 32.4–41.5 μM) against MCF-7, MDA-MB-231 and Caco-2 cancer cells (Wang et al. 2014). Compounds, fusaraisochromenone (85), fusaraisochromanone (86), (R)-3,4-dihydro-4,8-dihydroxy-6-methoxy-4,5-dimethyl-3-methyleneisochromen-1-one (87), 8-O-methyljavanicin (88), 3-acetyl-7-hydroxy-5-methoxyl-3H-isobenzofuran-l-one (89), curvulin (90), fusalanipyrone (91), daidzein (92), formononetin (93), 7-O-methyl genistein (94), kakkatin (95), p-hydroxy benzoic acid (96), and tyrosol (97) isolated from endophytic fungus *Fusarium* sp. PDB51F5 were reported to exhibit cytotoxic activity against various cell line with IC_50_ ranging from 148–162µM (Boonyaketgoson et al. 2015).

A new oxysporidinone analogue and a new 3-hydroxyl-2-piperidinone derivative, along with (−)-4,6′-anhydrooxysporidinone, (+)-fusarinolic acid, gibepyrone D, beauvercin, cerevisterol, fusaruside, and (2S,2′R,3R,3′E,4E,8E)-1-O-D-glucopyranosyl-2-N-(2′-hydroxy-3′-octadecenoyl)-3-hydroxy-9-methyl-4,8-sphingadienine isolated from *F. oxysporum* were evaluated for cytotoxicity against PC-3, PANC-1, and A549 cancer cell lines and only beauvericin (21) showed potency with IC_50_ ranging from 10.4 to 49.5μM (Wang et al. 2011). Fusarithioamide A (49) and ergosta-7,22-diene-3β,5α,6β-triol (98) produced by *F. chlamydosporium* endophyte of *Anvillea garcinii* were evaluated for their *in vitro* cytotoxic activity against KB, BT-549, SK-MEL, and SKOV-3 cell lines. The results showed that compound 98 was active towards all tested cell lines while, compound 49 possessed potent and selective activity towards BT-549 and SKOV-3 cell lines (Ibrahim et al. 2016a). Fusarithioamide B (50) isolated from the same extract was strongly potent and selective towards BT-549, MCF-7, SKOV-3, and HCT-116 cell lines with IC_50_ values of 0.09, 0.21, 1.23, and 0.59µM, respectively (Ibrahim et al. 2018b). These data suggest that fusarithioamide A and B could be a good starting point to provide promising anticancer candidate molecules for drug development.

The antibiotic compounds enniatins A1 (53), B (23) and B1 (52) isolated from *F. tricinctum*, an endophyte of *Aristolochia paucinervis* were reported to exhibit moderate cytotoxic activity against HepG2 and C6 cells (IC_50_ 10–25 µM), and high toxicicity against H4IIE cells (IC_50_ 1–2.5 µM). Additional mechanism of action study showed that all enniatins increased caspase 3/7 activity and nuclear fragmentation as markers for apoptotic cell death in H4IIE cells. Specifically, enniatin A1, B1, and, also enniatin B decreased the activation of extracellular regulated protein kinase (ERK) (p44/p42). Enniatins A1 and B1, was also able to inhibit moderately the activation of tumour necrosis factor a (TNF-a)-induced NF-jB (Wätjen et al. 2009). In a similar study, Vasundhara et al. (2016) showed that extract of *F. tricinctum*, an endophytic fungus of *Taxus baccata* was cytotoxic (IC_50_ 225–220μg/mL) against human breast cancer cell line (MCF-7), human cervical cancer cell line (HeLa) and peripheral blood mononuclear cells (PBMCs). Interestingly, this extract inhibited the proliferation of concanavalin A-stimulated PBMCs and the tumour necrosis factor (TNF)-α production in concanavalin A-stimulated PBMCs and MCF-7. These results are indicating that the antiproliferative activity observed could be associated with TNF-α and suggest that the chemical investigation of this extract could lead to the characterisation of potent anticancer compounds with the potential to inhibit the TNF-α.

Hemphill et al. (2017) by using the OSMAC (One Strain Many Compounds) approach, significantly increased (up to 80-fold) the production of fusarielin J (99), in addition to inducing the production of fusarielin K (100), fusarielin L (101), fusarielins A (102) and B (103) by *F. tricinctum*, endophyte from *Aristolochia paucinervis*. More interestingly, these compounds were active (IC_50_ 12.5–84.6μM) against the human ovarian cancer cell line A2780. This method consisting on manipulating culture conditions such as carbon, salts and minerals sources, temperature, pH, incubation time and many other parameters have been previously applied with success to induce the production of several bioactive metabolites (Bode et al. 2002).

## 6. Antioxidant and antiaging activities of metabolites from fusarium species

**Figure 5.**
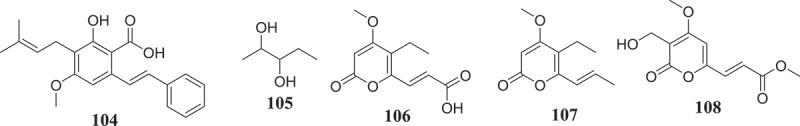
Antioxidant and antiaging compounds reported from *Fusarium* spp.

Gradually, the ability of endophytic fungi to produce potent antioxidant metabolites is been recognized. These bioactive compounds are often identified from screening using various antiradical and antioxidant assays (Figure 5). For instance, using the DPPH radical scavenging assay, Hamzah et al. (2018) reported the free radical scavenging potential of methanol-extract of *F. lateritium* endophyte of *Rhizophora mucronata*. Similarly, the phenolic content (160.51 mg of GAE/g of extract) and the free radical scavenging activity (IC_50_ 89.61 µg/mL) of ethyl acetate extract of *F. oxysporum* isolated from the flower part of *Dendrobium lindleyi* was also reported (Bungtongdee et al. 2019). Likewise, the free radical scavenging activity (IC_50_ 482 μg/mL) of crude metabolites from endophytic *F. tricinctum* was reported by Vasundhara et al. (2016). According to Khan et al. (2018), bostrycoidin (12), anhydrofusarubin (13), 4-hydroxybenzaldehyde (78) and fusarubin (7) exhibited significant antioxidant activity with IC_50_ values of 1.6, 12.4, 28.9 and 34.8 μg/mL, respectively. Another natural antioxidant compound named Cajaninstilbene acid (104) was reported from extracts of *F. solani* (ERP-07), *F. oxysporum* (ERP-10), and *F. proliferatum* endophytes of *Cajanus cajan* (Zhao et al. 2012). These studies have demonstrated the potential of *Fusarium* spp. to produce antioxidant compounds and could serve as encouragement for further investigations in order to identify more compounds that could be developed as novel antioxidant drugs.

According to Bungtongdee et al. (2019), extract from *F. oxysporum* with strong antimutagenic activity was found to be composed of gibepyrone A, pyrrolo [1, 2-a] pyrazine-1,4-dione, hexahydro-3-(2-methylpropyl) and indoleacetic acid as major components. Another study by Tiwari et al. (2014) reveals that as compared to 1,5-pentanediol, 2,3-pentanediol (105) isolated from *F. oxysporum* endophyte of *Curcuma amada* was found to improve antiaging properties against *Caenorhabditis elegans*. This compound structurally like the commercially available 1,5-pentanediol can be further developed as a new antiaging agent (Figure 5). This finding also suggests that more investigation into extracts from *Fusarium* species might lead to the identification of more compounds with antiaging potential.

Sperm motility and hyperactivation (HA) are important for fertility. Recently, there has been significant progress in understanding factors controlling these events such as the generation, and regulation of calcium signals. Both pH-regulated membrane Ca^2+^channels and Ca^2+^ stores have been implicated in controlling HA (Alasmari et al. 2013). Impressively, endophytic fungi from *Fusarium* spp. have been reported to produce compounds (Figure 5) such as (E)-3-(5-ethyl-4-methoxy-2-oxo-2H-pyran-6-yl) acrylic acid (106), cladobotrin V (107), allantopyrone A (80) and islandic acid-II methyl ester (108) with the ability to restore fertility (Shiono et al. 2014).

## 7. Immunosuppressive and immunomodulator metabolites from fusarium species

**Figure 6.**
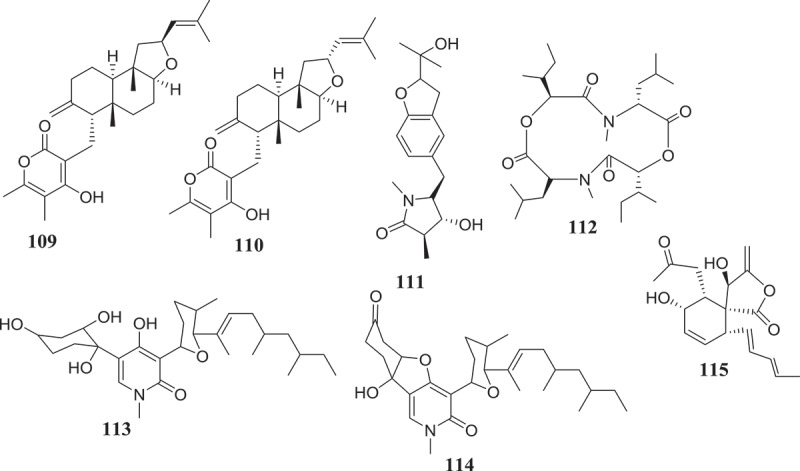
Metabolites with ability to influence the immune isolated from *Fusarium* spp.

Endophytic *Fusarium* species can also produce substances that can influence the immune system of animals (Figure 6). In reference to subglutinol A (109) and B (110), two well-known immunosuppressive agents produced by *F. subglutinans*, an endophyte of *T. wilfordii* (Lee et al. 1995), we may hypothetized that further investigation of extracts from *Fusarium* spp. might lead to the identification of compounds useful in the treatment of patients undergoing organs transplantation to avoid rejection.

Four compounds including, rigidiusculamide E (111), [-(*α*-oxyisohexanoyl-*N*-methyl-leucyl)_2_-] (112), (−)-oxysporidinone (113) and (−)-4,6′-anhydrooxysporidinone (114) isolated from *F. tricinctum* SYPF 7082, an endophyte of root of *P. notoginseng* were reported by Sun et al. (2018) to exhibit immunomodulatory activity. The similar potential was observed with Fusaspirol A (115) a new compound isolated from *F. solani* B-18 by Ariefta et al. (2019).

## 8. Antithrombotic agents from fusarium species

Cardiovascular diseases, such as acute myocardial infarction, ischaemic heart disease, and high blood pressure, are the leading causes of death in the world (Mine et al. 2005). Thrombin-mediated fibrinogen conversion to fibrin and fibrin monomer cross-linking result in the formation of a clot and the thrombosis occurs when the clots are not lysed. A variety of plasminogen activators have been widely studied and used as thrombolytic agents. However, these agents are very expensive and have side effects including gastrointestinal bleeding, and immunogenicity (Shao 2012). Therefore, new thrombolytic agents are in urgent demand. Although, several potential thrombolytic agents confirmed in clinical trials (Han et al. 2010), there is still an urgent need to explore new sources of fibrinolytic agents which could have better efficacy, specificity, and fewer side effects. Microbes are the most preferred source of such enzymes due to their diversity, feasibility in mass culture and ease in genetic manipulation (Mander et al. 2011). For the continuing search for antithrombotic agents from microbes, Wu et al. (2009) screened extracts from 1,075 endophytic fungi and identified extract from *Fusarium* sp. as the most potent. From that extract, an antithrombotic agent was identified as a 28-kDa single-chain fibrinolytic enzyme with no homology with other known fibrinolytic enzymes. From another study, a strongly potent fibrinolytic enzyme exhibiting not only protease activity between pH 2.5 and 11.5 but was also stability at high temperature (up to 50°C) was isolated from *Fusarium* sp. BLB isolated from leaves of Hibiscus. Additionally, the N-terminal amino acid sequence of this enzyme was identical to previously reported proteases (Ueda et al. 2007). These data are supporting the claims that fungi can be a good source for a fibrinolytic enzyme with potential for application in the treatment of thrombosis (Peng et al. 2005; Rovati et al. 2010; Zhang et al. 2015b).

## 9. Biocontrol ability of endophytic fusarium species

### 9.1 Induction of plant defence system

The potential of *Fusarium* endophytes to impact plant physiology and improve plant defence system have been intensively investigated. For instance, in tomato plant infected by *Tetranychus urticae*, the inoculation of endophyte *F. solani* strain K (FsK) isolated from the root tissues of tomato altered the plant responses by instigating the defence-related genes (Pappas et al. 2018). Additionally, Garantonakis et al. (2018) reveals that all plants colonized by FsK were more resistant to damage caused by *Nesidiocoris tenuis*. Likewise, another strain of *F. solani* was able to induce systemic resistance in tomato plants infected by *Septoria lycopersici* via the induction of pathogenesis-related genes (Kavroulakis et al. 2007). In *Hordeum vulgare*, the presence of endophyte *F. oxysporum* was able to confer disease resistance against virulent pathogens (Schulz et al. 1999). This resistance was positively correlated to the increasing concentrations of phenolic metabolites in the plant. This observation was supported by Yong et al. (2009) who correlated the enhancement in terpenoids concentration to the growth and resistance of *Euphorbia pekinensis* after inoculation of endophytic fungi *Fusarium* spp. Conjointly, these studies suggest that the presence of endophytes in host tissues can definitely help the plant to resist against invading pathogens not only by eliciting the host response mechanism, but also stimulating the production of antagonistic metabolites (Kloepper and Ryu 2006; Van Bael et al. 2012). These positive results may suggest that *Fusarium* species can effectively be harnessed as an effective tool to improve the resistance of crop plants to pathogens attack. Far more investigations in this area are needed to turn these species into biocontrol agents to fight against plant pathogenic microorganisms affecting the agricultural crop production industry.

### 9.2 Improve plant resistance against abiotic stresses

In addition to their ability to maintain the health of plants, *Fusarium* endophytes also play an imperative role in preparing the plant against abiotic stresses as well as enhancing the growth and the production yields (Lata et al. 2018). For instance, the presence of *F. culmorum* in *Leymus mollis* was found to increase his ability to tolerate the stress associated with high salinity (Rodriguez et al. 2008). In more recent study, Shah et al. (2019) demonstrated that endophytic *Fusarium* sp. from the root of *Dendrobium moniliforme* was able to promote the growth and development of *Rhynchostylis retusa*. This supported the finding of Cui et al. (2018) who reported earlier the ability of three endophytes isolates of *F. redolens* KY379544, *F. nematophilum* KY379572 and *F. nematophilum* KY379764 to promote the germination of *C. songaricum* seeds. Moreover, metabolites produced by several *Fusarium* spp. were also reported to promote the growth of plants. To name but a few, study by Zhong et al. (2016) showed that polysaccharides obtained from endophytic *F. oxysporum* stimulated the sprout growth, flavonoid accumulation, and antioxidant capacity of tartary buckwheat. Another *F. oxysporium* Dzf17 endophyte of *Dioscorea zingiberensis* was reported to induce the diosgenin accumulation in cell suspension culture of the host plant (Li et al. 2011).

It is clear that endophytes are an integral part of plant biology and served as important components of plant micro-ecosystems (Christian et al. 2016; Jia et al. 2016). Therefore, the concept of “mycovitalism” introduced by Vujanovic and Vujanovic (2007) after observing the positive effect of *F. semitectum* on orchid seed germination in increasingly accepted. Indeed, recent research data are leaning towards the holobionts nature of plants (Vandenkoornhuyse et al. 2015) and increasingly providing evidence that plants are dependent on the microbes inhabiting inside them (Selosse et al. 2014). All the evidence are showing today that endophytes, especially fungal endophytes play important beneficial roles in host plant development and physiology including increased stress tolerance, enhanced root growth and provision for special nutrition and water (Wani et al. 2015). This is also true for *Fusarium* species frequently isolated as an endophyte in a wide-varied host (Rather et al. 2018; Strobel 2018). Data presented above are supporting the ability of *Fusarium* species as an important component of the host plant and confirmed the potentiality of using these endophytic species to limit the effect of abiotic stresses on crop plants and improve the production yield.

### 9.3 Nematicidal activity

**Figure 7.**
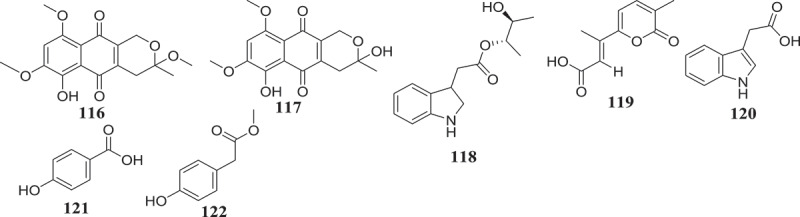
Nematicidal compounds produced by *Fusarium* spp.

In order to replace biohazardous nematocides, there is a strong drive of finding natural product-based alternatives to contain nematode pests affecting agricultural crops. The use of microorganisms in controlling plant-parasitic nematodes is receiving increasing attention as an important alternative for toxic chemicals. In this respect, endophytic fungi from *Fusarium* genus were investigated for their nematicidal potential and the data collected so far shows that these species may constitute a potential source for new nematicidal agents. In fact, several studies were reported describing the potential of endophytic *F. oxysporum* isolates to inhibit various nematodes parasites at different stages of their development (Hallman and Sikora 1994; Vu et al. 2004; Dubois et al. 2004; Mennan et al. 2005; Athman et al. 2006; Shahasi et al. 2006; Dababat and Sikora 2007; Mwaura et al. 2009; Mwaura et al. 2010; Yang et al. 2012; Van Dessel et al. 2011; Martinuz et al. 2012; Waweru et al. 2014). Another endophyte species, *F. moniliforme* strain Fe14 was also reported for his activity against *M. graminicola* by Le et al. (2016). This antagonist activity is often suspected to be achieved via the production of antinemic metabolites (Figure 7).

In fact, endophytic *F. oxysporum* exhibiting nematocidal activity was found to produce compounds bikaverin (67), 3-O-methyl-8-O-methyl fusarubin (116), 8-O-methyl fusarubin (117), anhydrofusarubin (13), and fusarubin (7), with activity against *M. incognita* (Kundu et al. 2016). Similarly, chlamydosporin (118) produced by *F. chlamydosporum*, isolated from the root of *Suaeda glauca* exhibited significant phytotoxic activity against the radicle growth of *Echinochloa crusgalli* (Wang et al. 2018). Four other compounds including gibepyrone D (119), indole‐3‐acetic acid (120), 4‐hydroxybenzoic acid (121) and methyl 2‐(4‐hydroxyphenyl) acetate (122), produced by *F. oxysporum* 162 (Fo162) displayed good nematocidal activities. Moreover, compound 120 was also found to trigger the plant resistance mechanism (Bogner et al. 2016). The overall presentation of data is demonstrating the outstanding biocontrol potential of *Fusarium* spp. and support the continue investigation of these fungi as sources of potential biocontrol agents.

## Conclusion and perspectives

Fungi belonging to *Fusarium* genus represent one of the most important groups of fungi because of their implication as plant pathogens. Fortunately, the non-pathogenic species from this genus, particularly endophytes are of equal importance because of their outstanding biosynthetic ability. In fact, our study demonstrates that endophytic *Fusarium* species are untapped bioresource with novel and interesting biological and chemical diversity. As a matter of fact, more than 100 structurally unique chemical compounds exhibiting a wide range of biological activities including antimicrobial, anticancer, antiviral, antioxidants, antimalarial, antiparasitics, immunosuppressants, immunomodulators, antithrombotic, and biocontrol have been reported from these species. However, this study also showed that only a few species from this large genus have been investigated so far, suggesting that a significant number of strains remain unexplored. Therefore, systematic bioguided investigations are needed to explore the full potential and the diversity of bioactive metabolites produced by *Fusarium* species. Investigating metabolites produced by these microorganisms can offer an opportunity to discover novel natural products with unique chemical scaffolds as starting points for lead discovery for the treatment of human and plant diseases.
